# Supplementary material for: Identification of photoperiod-induced specific miRNAs in the adrenal glands of Sunite sheep (Ovis aries)
Source: Front Vet Sci. 2022 Jul 22;9:888207. doi: 10.3389/fvets.2022.888207 (PMC9354845; doi:10.3389/fvets.2022.888207)
Supplement: Supplementary file 1 [file Data_Sheet_1.ZIP › Supplementary file/miRDeep2 file.zip!/miRDeep2 file/Novel_Structure/LP42A3/Novel_438.pdf]

Provisional ID	:	NC_019460.2_5582
Score total	:	1.9
Score for star read(s)	:	-1.3
Score for read counts	:	-1.3
Score for mfe	:	3
Score for randfold	:	1.6
Score for cons. seed	:	
Total read count	:	9
Mature read count	:	6
Loop read count	:	0
Star read count	:	3

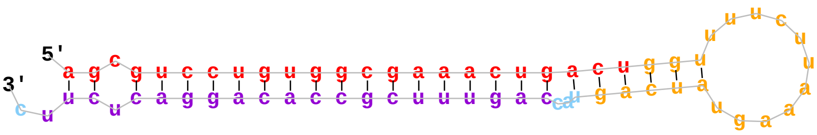

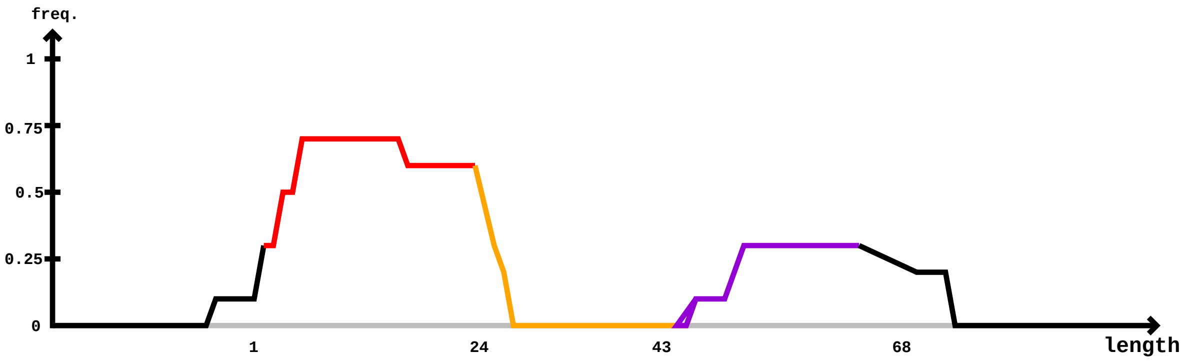

Mature   Star

```
5'- aagcugaggccuggguggga agcguccuguggcgaaacugacu gguuuucuuaaaguaucaguac caguuucgccacaggacucuu cccacccaguccucagcuuuaucuugg   -3'    obs
    aagcugaggccuggguggga agcguccuguggcgaaacugacu gguuuucuuaaaguaucag uaccaguuucgccacaggacucuuc ccacccaguccucagcuuuaucuugg          exp
    (((((((((.((((((((((((.(((((((((((((((((((((((...........))))))..))))))))))))))))))).)))))))))).)))))))))........   reads   mm   sample
    ...............ugggaagcguccuguggcga..............................................................................   1   0   seq
    ....................agcguccuguggcgaaacugacu......................................................................   2   0   seq
    ......................cguccuguggcgaaacugacu......................................................................   1   0   seq
    ......................cguccuguggcgaaacugacugg....................................................................   1   0   seq
    ........................uccuguggcgaaacugacuggu...................................................................   2   0   seq
    .................................................................caguuucgccacaggacucuu...........................   1   0   seq
    .....................................................................uucgccacaggacucuucccacc.....................   1   0   seq
    ......................................................................ucgccacaggacucuucccacc.....................   1   0   seq
```
